# EF24 induces ROS-mediated apoptosis *via* targeting *thioredoxin reductase 1* in gastric cancer cells

**DOI:** 10.18632/oncotarget.7633

**Published:** 2016-02-23

**Authors:** Peng Zou, Yiqun Xia, Weiqian Chen, Xi Chen, Shilong Ying, Zhiguo Feng, Tongke Chen, Qingqing Ye, Zhe Wang, Chenyu Qiu, Shulin Yang, Guang Liang

**Affiliations:** ^1^ School of Environmental and Biological Engineering, Nanjing University of Science and Technology, Nanjing, Jiangsu, China; ^2^ Chemical Biology Research Center, School of Pharmaceutical Sciences, Wenzhou Medical University, Wenzhou, Zhejiang, China; ^3^ Department of Digestive Diseases, The First Affiliated Hospital of Wenzhou Medical University, Wenzhou, Zhejiang, China

**Keywords:** thioredoxin reductase 1, reactive oxygen species, EF24, ER stress, gastric cancer

## Abstract

Gastric cancer (GC) is one of the leading causes of cancer mortality in the world, and finding novel agents for the treatment of advanced gastric cancer is of urgent need. Diphenyl difluoroketone (EF24), a molecule having structural similarity to curcumin, exhibits potent anti-tumor activities by arresting cell cycle and inducing apoptosis. Although EF24 demonstrates potent anticancer efficacy in numerous types of human cancer cells, the cellular targets of EF24 have not been fully defined. We report here that EF24 may interact with the thioredoxin reductase 1 (TrxR1), an important selenocysteine (Sec)-containing antioxidant enzyme, to induce reactive oxygen species (ROS)-mediated apoptosis in human gastric cancer cells. By inhibiting TrxR1 activity and increasing intracellular ROS levels, EF24 induces a lethal endoplasmic reticulum stress in human gastric cancer cells. Importantly, knockdown of TrxR1 sensitizes cells to EF24 treatment. *In vivo*, EF24 treatment markedly reduces the TrxR1 activity and tumor cell burden, and displays synergistic lethality with 5-FU against gastric cancer cells. Targeting TrxR1 with EF24 thus discloses a previously unrecognized mechanism underlying the biological activity of EF24, and reveals that TrxR1 is a good target for gastric cancer therapy.

## INTRODUCTION

Natural products and their derivatives have historically been invaluable as a source of therapeutic agents, and served human kind in the treatment of various diseases for centuries [1, 2]. It is roughly estimated that half of modern marketed drugs originate from natural products [3]. Thus, the past decades have witnessed increasing interest in identifying the active chemical components and their cellular targets, which leads to discovering numerous new therapeutic agents, such as vinblastine, rapamycin, and paclitaxel.

As an excellent natural compound, curcumin has been extensively investigated for its potential biological benefits, including anti-tumor [4], anti-inflammation [5], and anti-virus [6]. Moreover, structural curcumin analogs have been designed to optimize the therapeutic effects of curcumin by enhancing potency, reducing side-effects, and increasing bioavailability [7-9]. Diphenyl difluoroketone (EF24), a new compound having close structural similarity to curcumin, has been reported to inhibit the proliferation of a variety of cancer cells *in vitro* and *in vivo* [10, 11]. Due to the dual advantages in both pharmacology and pharmacokinetics, EF24 received much attention from pharmacologists in the world. It is one of curcumin analogs most close to anti-cancer clinical study. A number of molecular targets in various types of cells, such as NF-κB [12], p53 [13], HIF-1α [11, 14], AKT [10], mitogen-activated protein kinase family [15], and phosphatase and tensin homolog deleted on chromosome (PTEN) [16], have been reported to be affected by EF24. Despite its undoubted anticancer efficacy, the molecular mechanism underlying the action of EF24 still elusive, and the primary cellular target and mode of action of this molecule remain unclear.

The mammalian thioredoxin reductases (TrxRs) are a family of selenium-containing pyridine nucleotide-disulphide oxidoreductases. There are currently two confirmed forms of mammalian TrxRs, TrxR1 and TrxR2, which are found in cytoplasm and mitochondria, respectively [17, 18]. TrxR1 was overexpressed in many human tumors and has a key role in regulating intracellular redox balance [19-21]. TrxR1 inactivation by chemical inhibition or small interfering RNA (siRNA)-mediated knockdown inhibits self-sufficiency of tumor cells, reverts the malignant phenotype, and sensitizes tumor cells to chemotherapeutic drugs [22-24]. Hence, TrxR1 has emerged as a valuable target for anticancer drug development [25, 26].

In the present study, we discovered that EF24 may interact with TrxR1 both *in vitro* and *in vivo*. EF24 primarily targets the Sec residue of the antioxidant enzyme TrxR1 to inhibit its Trx-reduction activity, but further elicits a new function of generating reactive oxygen species. Accumulation of ROS disrupts the intracellular redox balance, activates ER-stress and eventually induces apoptosis in gastric cancer cells. Knockdown of TrxR1 in cells enhances the cyto-toxicity of EF24. Remarkably, EF24 treatment significantly reduces the TrxR1 activity and tumor cell burden *in vivo*. In addition, EF24 displays synergistic lethality with 5-FU *in vivo*. Targeting TrxR1 by EF24 thus reveals an unprecedented mechanism underlying the biological action of EF24 and sheds deep light on the potential application of EF24 in the treatment of cancer.

## RESULTS

### EF24 treatment selectively kills human gastric cancer cells but not normal cells

**Figure 1:**
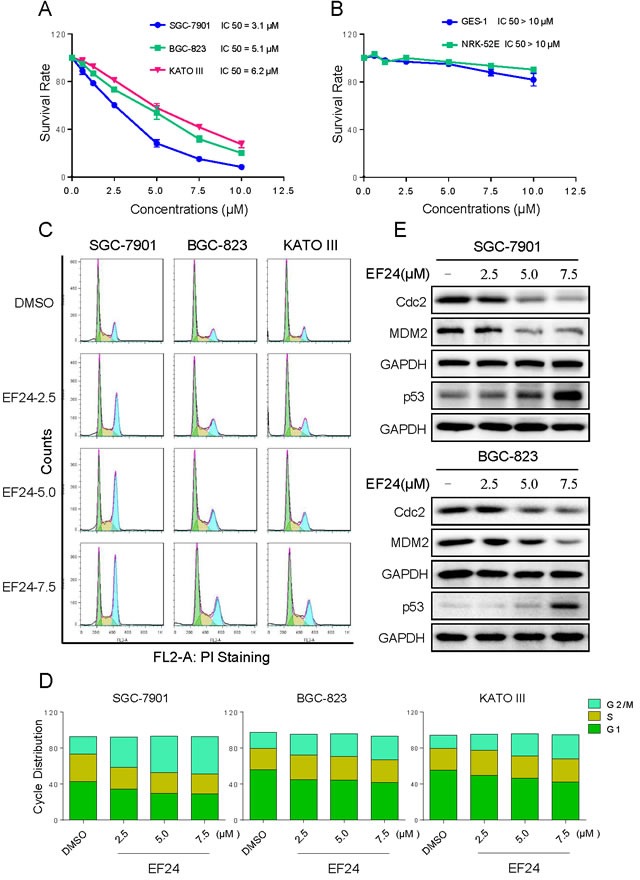
EF24 treatment selectively kills human gastric cancer cells but not normal cells **A.**-**B.** The effects of EF24 on the proliferation of human gastric cancer cell lines and normal cell lines. SGC-7901, BGC-823, KATO III, GES-1 or NRK-52E cells were incubated with increasing doses of EF24 (0.625-10 μM) for 24 h respectively. Cell viability was determined by MTT assay. **C.** Induction of cell cycle arrest in human gastric cancer cells was determined by flow cytometry after treatment with EF24 (2.5, 5.0 or 7.5 μM) for 14 h. Similar results were obtained in three independent experiments. **D.** Representative histograms from flow cytometry analysis in three human gastric cell lines treated with EF24 (*p < 0.05, **p < 0.01). **E.** Expression of G2/M cell cycle relative proteins Cdc2, MDM2 and p53 were determined by western blotting after treatment with EF24 for 14 h.

The cytotoxic effects of EF24 were tested in cultured human gastric cancer cells and normal cells. EF24 treatment markedly inhibited the growth of gastric cancer cells, while the viability of normal GES-1 and NRK-52E cells were affected only minimally at the highest concentration (10 μM) tested (Figure 1A-1B). To determine whether the growth inhibition of gastric cancer cells by EF24 was caused by cell cycle arrest, gastric cells were treated with various concentrations of EF24 for 14 h. The results in Figure 1C and 1D showed that EF24 treatment significantly induced G2/M cell cycle arrest in human gastric cancer SGC-7901, BGC-823 and KATO III cells. Western blotting analysis indicated that EF24 treatment also dose-dependently inhibited the expression of cell cycle-related proteins such as MDM2 and Cdc2 in these gastric cancer cells. In contrast, p53 was significantly increased with EF24 treatment (Figure 1E). These data suggest that the inhibition of cell proliferation by EF24 is partly associated with the induction of G2/M phase arrest.

### EF24 induced apoptosis in human gastric cancer cells

**Figure 2:**
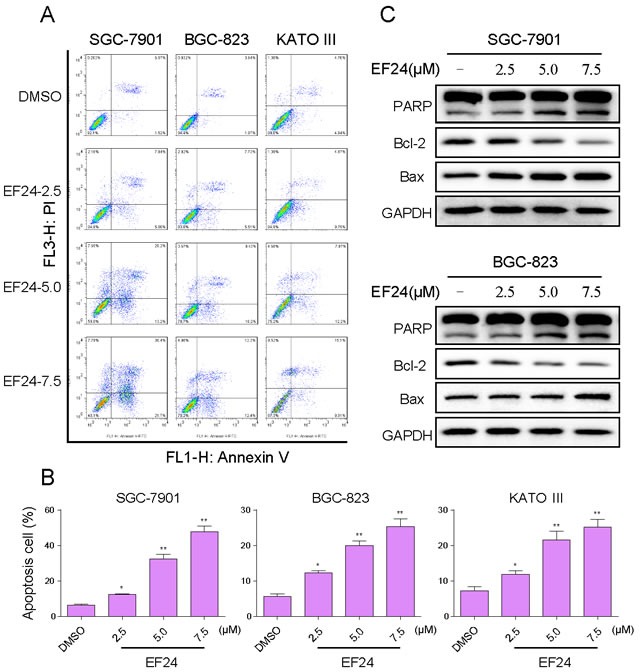
EF24 induced apoptosis in human gastric cancer cells **A.** Induction of apoptosis in human gastric cancer cells was determined by flow cytometry after treatment with EF24 (2.5 5.0 or 7.5 μM) for 24 h. Similar results were obtained in three independent experiments. **B.** The percentage of apoptotic cells in the treatment groups was calculated (* *p*< 0.05, ** *p* < 0.01). **C.** SGC-7901 and BGC-823 cells were treated with EF24 (2.5, 5.0 or 7.5 μM) for 20 h. Whole-cell lysates were subjected to western blotting to assess the expression of cell apoptosis related proteins. GAPDH was used as internal control.

We further examined the pro-apoptosis effect of EF24 on human gastric cancer cells using Annexin V/Propidium Iodide (PI) staining assay. As shown in Figure 2A and 2B, all of three gastric cancer cell lines have shown a concentration-dependent apoptosis after a 24 h treatment with EF24. Then we determined the levels of apoptosis-related proteins in SGC-7901 and BGC-823 cells treated with EF24. Figure 2C showed that treatment with EF24 for 20 h dose-dependently increased the expression of cleaved-PARP and Bax, whereas Bcl-2 was downregulated compared with non-EF24-treated controls. These results suggest that the anti-cancer effect of EF24 is also associated with the induction of cell apoptosis.

### EF24 activates ER stress, which contributes to EF24 lethality in gastric cancer cells

**Figure 3:**
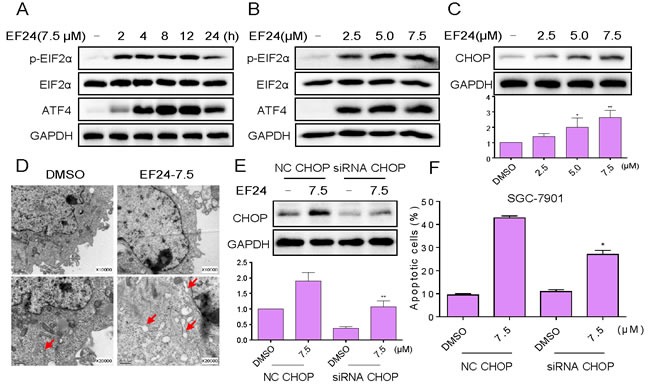
EF24 activates ER stress, which contributes to EF24 lethality in gastric cancer cells **A.** SGC-7901 cells were treated with EF24 (7.5 μM) for the indicated times, the protein levels of p-eIF2α and ATF4 were determined by western blot. **B.** SGC-7901 cells were treated with EF24 (2.5, 5 or 7.5 μM) for 3 h, the p-eIF2α and ATF4 expression were detected by western blotting. The protein level of CHOP was examined by western blot after treatment with EF24 for 12 h **C.**. GAPDH was used as internal control. **D.** Effect of EF24 on the morphology of endoplasmic reticulum in SGC-7901 cells. SGC-7901 cells were treated with EF24 (7.5 μM) for 3 h and the morphology of endoplasmic reticulum in SGC-7901 cells was examined with an electron microscope (×10000 or ×20000). Results from a representative cell sample out of three studied in each group are shown. **E.** SGC-7901 cells were infected with CHOP siRNA or control siRNA, CHOP expression in SGC-7901 cells was determined by western blotting after stimulation with EF24 (7.5 μM) for 12 h. **F.** SGC-7901 cells transfected with CHOP siRNA or control siRNA were treated with EF24 (7.5 μM) for 24 h. Percentage of cell apoptosis was determined by Annexin-V/PI staining and flow cytometry.

The next step is to investigate the underlying mechanisms of the anti-cancer effects of EF24. SGC-7901 cells were used for the subsequent studies. It is reported that ER stress plays an important role in the initiation of curcumin-induced apoptosis [27, 28]. Therefore, we hypothesize that exacerbation of ER stress contributes to gastric cancer cells apoptosis by EF24 treatment. We next examined the expressions of ER stress-related proteins, such as p-eIF2α and ATF4 in EF24-treated gastric cancer cells. The time-course result indicated that EF24 (7.5 μM) could significantly activates ER stress. The expression levels of p-eIF2α and ATF4 reached the peak at 3-6 h after treatment (Figure 3A). EF24 also showed dose-dependent activation of the two protein expressions in the gastric cancer cells (Figure 3B). CHOP induction is probably the most sensitive to ER stress response, and CHOP is considered as a marker of commitment of ER stress-induced apoptosis [29]. We found that EF24 treatment significantly induced CHOP protein expression in a dose-dependent manner (Figure 3C). To confirm the accumulation of misfolded proteins in ER, the effect of EF24 on the morphology of ER in SGC-7901 cells was observed through electron microscopy. Compared with DMSO-treated SGC-7901 cells, the ER in SGC-7901 cells after 3 h of treatment with EF24 (7.5 μM) became swelling (arrow), suggesting the accumulation of misfolded proteins in ER (Figure 3D). We next determined the effects of siRNA-mediated depletion of CHOP in gastric cancer cells. Knockdown of CHOP by siRNA, markedly attenuated CHOP expression in SGC-7901 cells (Figure 3E). This was associated with an appreciable reduction in EF24-induced apoptosis in SGC-7901 cells (Figure 3F). These findings demonstrate that EF24-induced cell apoptosis is at least partly mediated by ER stress pathway.

### ROS generation is the regulator of EF24-induced apoptosis

**Figure 4:**
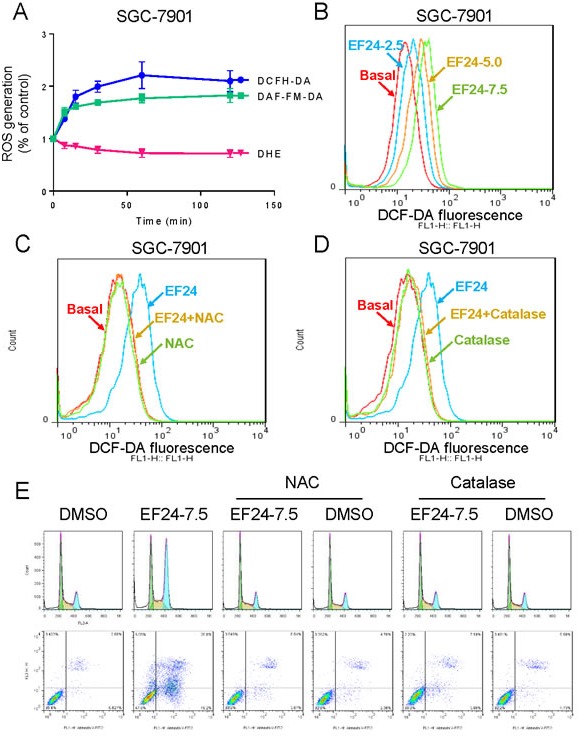
ROS generation is the key regulator of EF24-induced apoptosis **A.** Intracellular ROS generation induced by EF24 was measured in SGC-7901 cells by staining with DCFH-DA (10 μM), DAF-FM-DA (5 μM) or DHE (5 μM) and flow cytometry analysis. **B.** Intracellular ROS generation induced by increasing doses of EF24 was measured in SGC-7901 cells by flow cytometry. **C.**-**D.** SGC-7901 cells were pre-incubated with NAC or catalase for 2 h before exposure to EF24 (7.5 μM) for 1 h. Intracellular ROS generation was measured by flow cytometry. **E.**-**F.** Blocking of ROS generation abolished the cytotoxicity of EF24. SGC-7901 cells were pre-incubated with or without NAC or catalase for 2 h before exposure to EF24 (7.5 μM). Cell cycle arrest and percentage of cell apoptosis were determined by flow cytometry.

It has been recognized that reactive oxygen species (ROS) production in cancer cells triggered by some therapeutic agents is one of the mechanisms underlying their cytotoxicity [30-32]. Also, ROS overproduction has been reported to activate ER stress and mitochondrial apoptosis [33]. Therefore, we determined the production of intracellular ROS in EF24-treated and untreated cells by flow cytometry. As shown in Figure 4A, treatment with EF24 in SGC-7901 cells caused a time-dependent increase in DCF-reactive ROS. Using a series of fluorescent probes specific for individual species of ROS, we found that hydrogen peroxide and nitric oxide, but not superoxide anion, were among the ROS species induced by EF24 in gastric cancer cells. In addition, treatment with EF24 for 1 h in SGC-7901 cells caused a dose-dependent increase in DCF-reactive ROS (Figure 4B). To identify the role of ROS in mediating EF24's anti-cancer effects, ROS inhibitor NAC and catalase were used. Interestingly, it was found that co-treatment with NAC or catalase fully reversed the EF24-induced increase in ROS (Figure 4C-4D). Similar results were observed in the cell cycle arrest and apoptosis assay detected by flow cytometry (Figure 4E). These findings demonstrate that ROS generation is the key regulator of EF24-induced apoptosis.

### EF24 directly binds and inactivates TrxR1 in human gastric cancer cells

**Figure 5:**
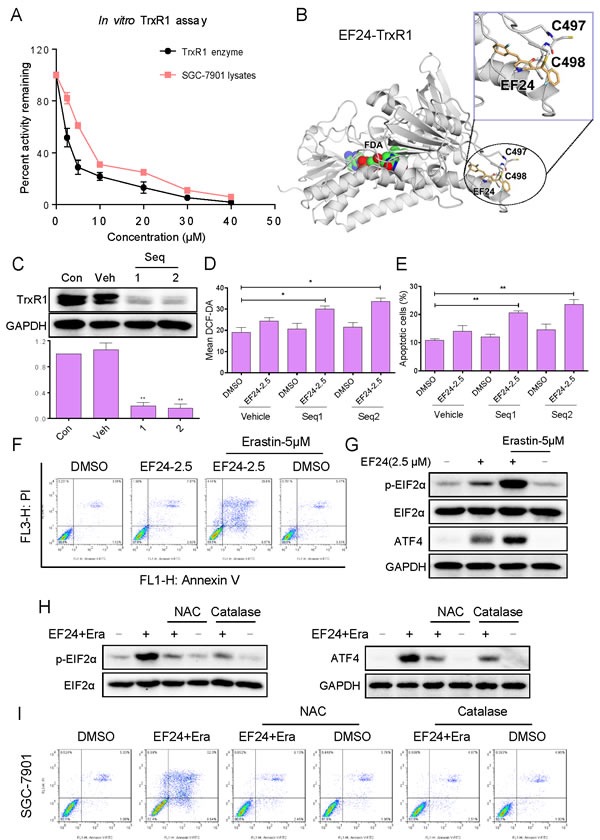
EF24 directly binds and inactivates TrxR1 in human gastric cancer cells **A.** TrxR1 enzyme activity was measured with/without EF24 treatment *in vitro*. **B.** Molecular docking of EF24 with TrxR1 protein was carried out with the docking software. **C.** The TrxR1 expression was determined by western blotting after knockdown with two different siRNAs for 48 h. **D.**-**E.** Knockdown of TrxR1 in SGC-7901 cells significantly increased the ROS levels **D.** and apoptotic cells **E.** induced by EF24. **F.** Dramatically augmentation of the EF24 cytotoxicity to gastric cancer cells by GSH depletion. **G.** EF24 and erastin in combination dramatically activated ER-stress pathway. **H.** NAC or catalase addition reversed combined treatment-induced expression of ER-stress related proteins. **I.** Blocking of ROS generation abolished the cytotoxicity of combined treatment.

The thioredoxin system is multifunctional redox active protein disulphide reductase systems, widely distributed in nature [34]. Accumulating evidence showed that ROS might be produced when the mammalian TrxR1 was chemically inhibited [30, 35]. Analysis of the chemical structure of EF24 reveals that this molecule belongs to a class of highly reactive organic compounds whose chemical structures (michael acceptor) allow them to interact with biological molecules by forming covalent bonds [36, 37]. Thus we speculated that EF24 might be a novel inhibitor of TrxR. Then, we evaluated the direct inhibitory effects of EF24 on TrxR1 enzyme activity by using the DTNB and the endpoint insulin reduction assay. When prereduced recombinant human TrxR1 was incubated with various concentrations of EF24 for 2 h, the DTNB-reducing activity of TrxR1 was decreased in a dose-dependent manner. To further explore the inhibition of TrxR1 by EF24, we determined the potency of EF24 at inhibiting the TrxR1 in SGC-7901 cells. Consistent with the inhibition of recombinant human TrxR1 protein, TrxR1 activity in cell lysates also decreased with increasing EF24 concentration (Figure 5A). Thus, it is confirmed that EF24 can inhibit TrxR1 enzyme activity *in vitro*. To further investigate the underlying structural mechanism of EF24 binding to the TrxR1 protein, we performed a molecular simulation of EF24-TrxR1 complex using docking software. As shown in Figure 5B, the michael acceptor of EF24 form a covalent bond with the residue Cys-498 of the C-terminal active site redox center of TrxR1. Additionally, EF24 interacts with the residues Cys-497 through the formation of hydrogen bonds. Thus, the proposed reaction mechanism for EF24 is to block simultaneously the adjacent C-terminal active site residues Cys and Sec of TrxR1, which is expected to effectively suppress TrxR1 activity.

Above data suggested the importance of TrxR1 in EF24-induced ROS production and cell apoptosis. To validate this note, genetic knockdown of TrxR1 was used in SGC-7901 cells. Knockdown of TrxR1 by two separate TrxR1 siRNAs showed that Seq1 and Seq2 markedly reduced TrxR1 expression in SGC-7901 cells (Figure 5C). The TrxR1 knockdown by either Seq1 or Seq2 resulted in an appreciable increase in EF24-induced ROS levels and apoptosis in SGC-7901 cells (Figure 5D and 5E). GSH is a pivotal component of the glutathione system, which is a redox regulation network in cells in addition to the thioredoxin system, and it also acts as a backup of the thioredoxin system [38]. The depletion of GSH by erastin in SGC-7901 cells significantly sensitizing the cells to EF24 supported the involvement of the thioredoxin system in the biological action of EF24 (Figure 5F). Abundant intracellular ROS may cause DNA damage and activate down-streamed signaling pathway. Therefore, it was interested to investigate whether the ER-stress pathway was activated by combined treatment. As shown in Figure 5G, treatment of cell with EF24 alone slightly induced the expressions of p-eIF2α and ATF4. However, EF24 and erastin in combination dramatically activated ER-stress pathway, as convinced by significantly enhanced expressions of p-eIF2α and ATF4. In addition, NAC and catalase pretreatment completely blocked the combined treatment-induced overexpression of p-eIF2α and ATF4 and apoptosis in SGC-7901 cells (Figure 5H and 5I). These results demonstrate that EF24 directly bound to TrxR1, and then increased cellular ROS and induced cancer cell apoptosis.

### EF24 inhibits SGC-7901 xenograft tumor growth *in vivo*, accompanied with decreased TrxR1 activity and increased ROS level

**Figure 6:**
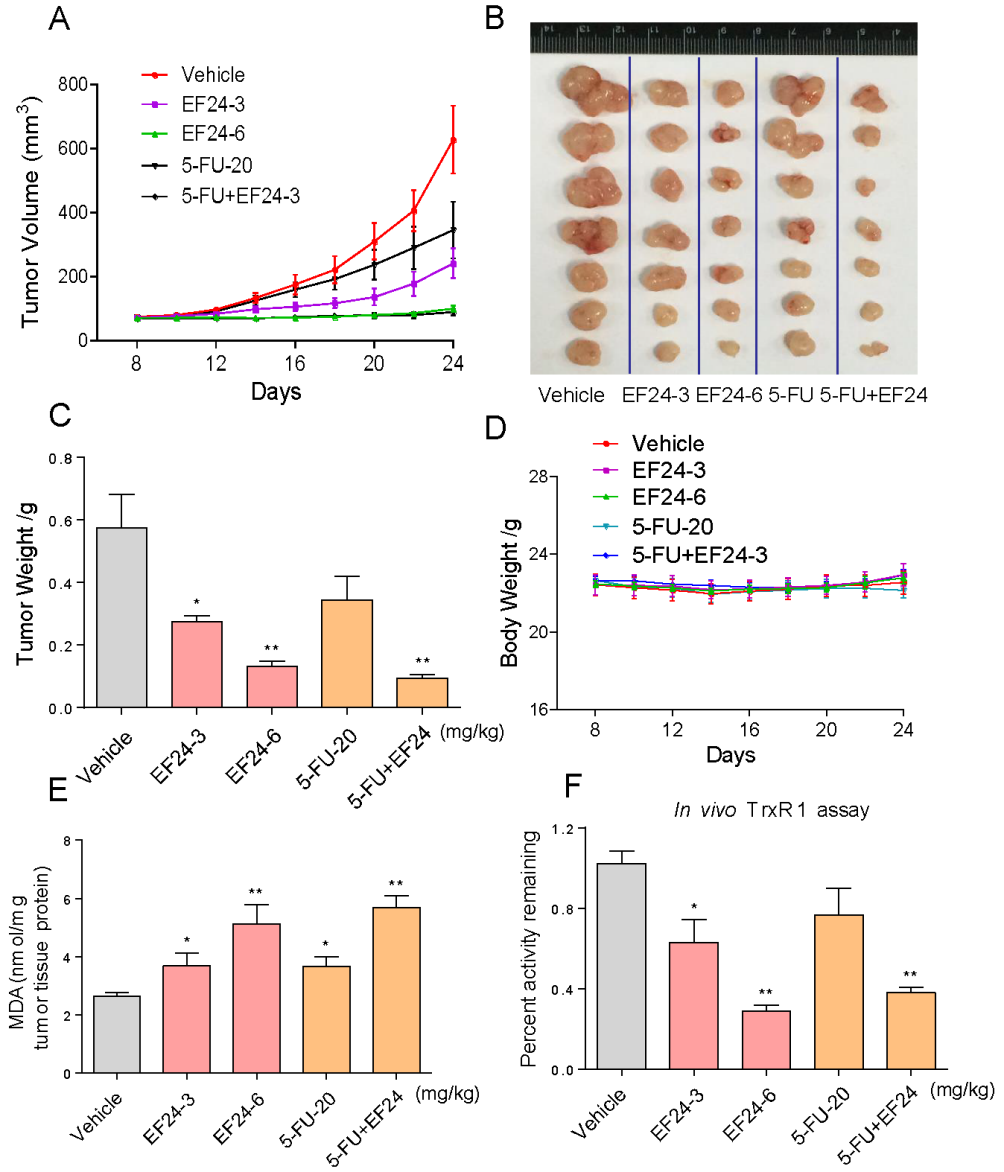
EF24 inhibits SGC-7901 xenograft tumor growth *in vivo*, accompanied with decreased TrxR1 activity and increased ROS level **A.**-**B.** EF24 treatment dose-dependently inhibited tumor volume and tumor weight **C.** of SGC-7901 human gastric cancer xenografts in nude mice, but did not affect body weight **D.** of mice. **E.** The levels of MDA in the tumor tissues. **F.** EF24 treatment dose-dependently inhibited the activity of TrxR1 *in vivo*. The tumor tissues were lysed and 100 μg of protein was used to determine the TrxR1 activity by using the endpoint insulin reduction assay. (A-F) EF24 enhances 5-FU-induced growth inhibition of tumor xenografts by targeting TrxR1 *in vivo*.

To evaluate the *in vivo* impact of EF24 treatment, we used a subcutaneous xenograft model of SGC-7901 cells in immunodeficient mice. Intraperitoneal administration of EF24 at doses of both 3 and 6 mg/kg for 17 days significantly reduced SGC-7901 tumor volume and weight versus vehicle control (Figure 6A-6C). Importantly, EF24 treatment for 17 days was well tolerated, without significant weight loss (Figure 6D). In addition, we found that EF24 dose-dependently increased the level of lipid peroxidation product (MDA), a marker of ROS, in tumor tissues (Figure 6E). TrxR1 activity in tumor xenografts was measured by the endpoint insulin reduction assay, and the result indicated that EF24 treatment significantly decreased the activity of TrxR1 in a dose-dependent manner (Figure 6F). Furthermore, EF24 remarkably amplifies the therapeutic effect of 5-fluorouracil (5-FU) *in vivo*, as convinced by significantly decreased tumor volume and weight (Figure 6A-6C). The *in vivo* mechanistic studies revealed that combined treatment inhibited tumor xenografts by inhibiting TrxR1 activity and activating oxidative stress (Figure 6E-6F). Taken together, these results indicate that EF24 exhibits potent anti-tumor activity and high safety *in vivo*, and EF24 can synergistically enhance 5FU-induced tumor growth inhibition *in vivo* by targeting TrxR1.

## DISCUSSION

Various chemotherapy drugs, including doxorubicin, 5-fluorouracil, and cisplatin have been used to treat gastric cancer. Unfortunately, all of these anticancer drugs affect not only pathological tumor cells, but also normal cells [39-41]. Therefore, the search for new chemopreventive and antitumor agents that are more effective but less toxic has become a matter of great interest. EF24, a new compound having close structural similarity to curcumin, has been reported to inhibit the proliferation of a variety of cancer cells *in vitro* and *in vivo* [11, 13, 15]. To date, the mechanism responsible for anti-cancer effects of EF24 remains elusive, and the primary cellular target of this molecule remain unclear. Our study show that EF24 treatment inactivates TrxR1, exacerbates endoplasmic reticulum stress and preferentially kills human gastric cancer cells. In addition, our data suggest that the strategy to use EF24 and 5-FU in combination could be a highly efficient way to achieve anticancer synergism by targeting TrxR1 *in vivo*.

The thioredoxin system has emerged as an important target in cancer chemotherapy, because both Trx1 and TrxR1 have been shown to be overexpressed in a variety of human cancer types and associated with increased tumor growth, drug resistance and poor patient prognosis [19, 23, 42, 43]. Thus, the past years have witnessed increasing attention to developing novel inhibitors of the system as potential antitumor agents. In the current study, we identified TrxR1 as a target of EF24 and demonstrated that EF24 induces apoptosis through a previously uncharacterized mechanism by targeting this enzyme. Binding of EF24 to TrxR1 inhibits the physiological functions of TrxR1, which leads to ROS accumulation within cells, consequently causing intracellular thiol depletion and finally eliciting oxidative stress. The glutathione and thioredoxin systems are two predominant networks that work independently but with some overlaps in maintaining the intracellular redox balance and defending against oxidative stress [44]. Recent studies indicate that the glutathione system can act as a backup of the thioredoxin system [38]. Interestingly, our results showed that downregulation of intracellular GSH by erastin significantly enhanced EF24's cytotoxicity. Combination therapy inhibiting both GSH and TrxR antioxidant pathways may yield success when applied in the clinical setting. Further insight into the roles of other antioxidants, and how they act both alone and together, will no doubt provide important clues into more effective treatments for cancer patients.

ER is also well known to regulate cellular responses to stress. Disturbance of ER homeostasis results in the activation of unfolded protein response. Accumulation of misfolded proteins in ER can cause ER stress and ultimately lead to apoptosis [45]. ER stress-induced cancer cell apoptosis becomes an important signaling target for development of cancer therapeutic drugs. The inductions of cancer cell apoptosis by some anti-cancer agents such as auranofin [35], farnesol [46] and arsenic trioxide [47] have been reported to be mediated by ER stress. Curcumin has also been found to activate ER stress in various cancer cell lines [27, 48]. In consistent with these findings, EF24 treatment concomitantly induces ER stress response, which is highlighted by elevated levels of p-eIF2α, and ATF4, as well as increase in the levels of CHOP. CHOP is considered as a marker of commitment of ER stress-induced apoptosis [29]. Findings presented here demonstrated that the siRNA-mediated knockdown of CHOP modestly inhibits EF24-induced apoptosis suggest that EF24-induced ER stress is a secondary response to EF24-induced ROS in gastric cancer cells. Collectively, our results underscore that EF24-induced oxidative stress is linked to proteotoxic and ER stress-based UPR, which can amplify the lethal effects of EF24 in gastric cancer cells.

In conclusion, we have discovered TrxR1 as a target of EF24, both *in vitro* and *in vivo*, and demonstrated that EF24 induces apoptotic cell death through ROS-mediated ER-stress. The observation that EF24 has synergistic cytotoxic effects with 5-FU sheds new light on the possibility of treating tumors with EF24 in combination with existing oxidative stress-causing anticancer drugs or physical treatments. The discovery of EF24-TrxR1 interaction provides deep insight into the understanding of how EF24 acts *in vivo* and this novel targeting mechanism may lead to the development of potent small-molecule TrxR1 inhibitors as potential cancer chemotherapeutic agents.

**Figure 7:**
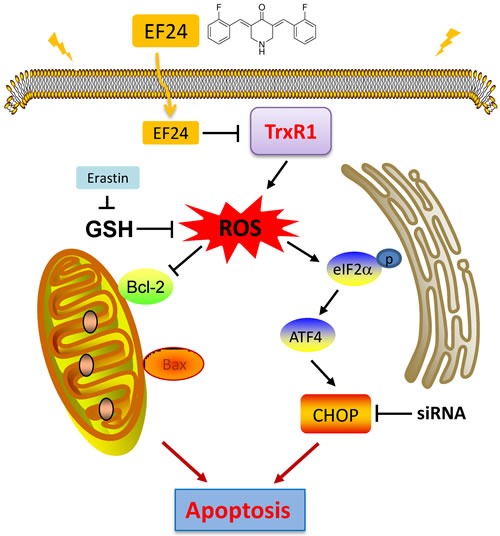
Schematic illustration of the underlying mechanism of EF24's anti-cancer activity

## MATERIALS AND METHODS

### Cell culture and reagents

EF24, N-acetylcysteine (NAC), catalase and rhTrxR1 protein were purchased from Sigma (St. Louis, MO). Erastin was purchased from Selleck Chemicals. Human gastric cancer cell lines SGC-7901, BGC-823 and KATO III, normal Human Gastric Epithelial Cell Line (GES-1) and normal Rat Kidney Proximal Cell Line (NRK-52E) were purchased from the Institute of Biochemistry and Cell Biology, Chinese Academy of Sciences. The cells were routinely cultured in RPMI 1640 medium (Gibco, Eggenstein, Germany) containing 10% heat-inactivated fetal bovine serum (Gibco, Eggenstein, Germany), 100 units/mL penicillin, and 100 μg/mL streptomycin in a humidified cell incubator with an atmosphere of 5% CO_2_ at 37°C. Antibodies including anti-Bcl-2, anti-Bax, anti-cleaved PARP, anti-TrxR1, anti-GAPDH, goat anti-mouse IgG-HRP and donkey anti-rabbit IgG-HRP were purchased from Santa Cruz Biotechnology (Santa Cruz, CA). Antibodies including anti-CHOP, anti-ATF4, anti-p-eIF2α and anti-eIF2α were purchased from Cell Signaling Technology (Danvers, MA). FITC Annexin V apoptosis Detection Kit I and Propidium Iodide (PI) were purchased from BD Pharmingen (Franklin Lakes, NJ).

### Cell viability assay

Cells were seeded into 96-well plates at a density of 8×10^3^ per well and allowed to grow overnight in RPMI 1640 containing 10% heat-inactivated FBS. EF24 was dissolved in DMSO and diluted with 1640 medium to final concentrations of 0.625, 1.25, 2.5, 5.0, 7.5 and 10 μM. The tumor cells were incubated with EF24 for 24 h before the MTT assay.

### Cell cycle analysis

Cells were placed on 60-mm plates for 12 h, and then treated with EF24 (2.5, 5.0, or 7.5 μM) for 14 h. The DNA was labeled with propidium iodide. Cell cycle analysis was performed in an FACS Calibur flow cytometer (BD Biosciences, CA).

### Cell apoptosis analysis

SGC-7901, BGC-823 and KATO III cells were plated on 60-mm dishes for 12 h, and then treated with EF24 (2.5, 5.0 or 7.5 μM) for 24 h. Cells were then harvested, washed twice with ice-cold PBS, and evaluated for apoptosis by double staining with FITC conjugated Annexin V and Propidium Iodide (PI) in binding buffer for 30 min using a FACSCalibur flow cytometer (BD Biosciences, CA).

### Measurement of reactive oxygen species generation

Cellular ROS contents were measured by flow cytometry as described previously [8]. Briefly, 5×10^5^ cells were plated on 60-mm dishes, allowed to attach overnight, and exposed to EF24 for the indicated time periods. Cells were stained with 10 μM DCFH-DA, 5 μM DAF-FM-DA or 5 μM DHE (Beyotime Biotech, Nantong, China) at 37°C for 30 min. Cells were collected and the fluorescence was analyzed using a FACSCalibur flow cytometer (BD Biosciences, CA). In some experiments, cells were pretreated with NAC or catalase for 2 h prior exposure to compounds and analysis of ROS generation.

### Western blotting analysis

Cells were homogenized in protein lysate buffer, and debris was removed by centrifugation at 12,000 g for 10 min at 4°C. The protein concentrations in all samples were determined by using the Bradford protein assay kit (Bio-Rad, Hercules, CA). After addition of sample loading buffer, protein samples were electrophoresed and then transferred to poly-vinylidene difluoride transfer membranes. The blots were blocked for 2 h at room temperature with fresh 5% nonfat milk in TBST and then incubated with specific primary antibody in TBST overnight at 4°C. Following three washes with TBST, the blots were incubated with horseradish peroxidase-conjugated secondary antibodies for 1 h, and the immunoreactive bands were visualized by using ECL kit (Bio-Rad, Hercules, CA). The density of the immunoreactive bands was analyzed using Image J computer software (National Institute of Health, MD).

### *In vitro* TrxR activity assays

The TrxR activity was determined by the DTNB assay at room temperature using a microplate reader (SpectraMax M5, Molecular Devices, USA). The NADPH-reduced TrxR (170 nM) was incubated with various concentrations of EF24 for 2 h at room temperature (the final volume of the reaction mixture was 50 μL) in a 96-well plate. A master mixture of TE buffer (50 mM Tris-HCl, pH 7.5, 1 mM EDTA, 50 μL) containing DTNB and NADPH was added (final concentrations: 2 mM and 200 μM, respectively), and the linear increase in absorbance at 412 nm during the initial 3 min was recorded. The same amounts of DMSO (1%, v/v) were added to the control experiments and the activity was expressed as the percentage of the control.

### Determination of TrxR activity in cells or tumor tissues

After cells were treated with various concentrations of EF24 for 2 h, the cells were harvested and protein extracted with RIPA buffer. The total protein content was determined by using the Bradford protein assay kit (Bio-Rad, Hercules, CA). TrxR activity in cell lysates or tumor tissues was measured by the end point insulin reduction assay. Briefly, the cell extract containing 100 μg of total proteins was incubated in a final reaction volume of 50 μL containing 100 mM Tris-HCl (pH 7.6), 0.3 mM insulin, 660 μM NADPH, 3 mM EDTA, and 15 μM E. coli Trx (Sigma, St. Louis, MO) for 30 min at 37°C. The reaction was terminated by adding 200 μL of 1 mM DTNB in 6 M guanidine hydrochloride (pH 8.0). A blank sample, containing everything except Trx, was treated in the same manner. The absorbance at 412 nm was measured, and the blank value was subtracted from the corresponding absorbance value of the sample. The activity was expressed as the percentage of the control.

### Docking of EF24 to the TrxR1 structural model

To further study the interaction between the EF24 and TrxR1, a covalent dock was implement by CovalentDock [49]. The crystal structure of human TrxR1 (PDB code 2ZZ0, chain A) was used for present docking study. The center coordination of dock pocket was set as −29.11, −1.26, and −6.55 which calculated by selecting residue Cys-497 and Cys-498. A grid box size of 60×60×60 points with a spacing of 0.375 Å between the grid points was implemented. The default parameters were used for running the docking simulation.

### Transient transfection of small interfering RNA (siRNA)

The sequences for the CHOP siRNA construct were described previously [8]. Small interfering RNA molecules, specifically targeting the TrxR1 mRNA, were obtained from Sigma (St. Louis, MO). For phenotypic confirmation, two different siRNA sequences were used (herein named Seq1 and Seq2). Seq1, sense 5′-(GCAAGACUCUCGAAAUUAU)dTdT-3′, antisense 5′-(AUAAUUUCGAGAGUCUUGC)dAdG-3′, Seq2, sense5′-(CUUUGCAGCUGCGCUCA AA)dTdT-3′, antisense 5′-(UUUGAGCGCAGCUGCAAAG)dTdT-3′. The siRNA duplexes targeting CHOP and TrxR1 were transduced into SGC-7901 cells. Forty-eight hours posttransduction, the cells were washed with complete media and plated with or without EF24 for 12 h for immunoblot analysis or 24 h for assessing apoptosis.

### Electron microscopy

SGC-7901 cells were treated with vehicle control (DMSO, 3 μL) or EF24 at the dose of 7.5 μM for indicated time in 60 mm plates. Then the cells were collected and fixed in phosphate buffer (pH 7.4) containing 2.5% glutaraldehyde overnight at 4°C. The cells were postfixed in 1% OsO4 at room temperature for 60 min, stained with 1% uranyl acetate, dehydrated through graded acetone solutions, and embedded in epon. Areas containing cells were block mounted and cut into 70 nm sections and examined with the electron microscope (H-7500, Hitachi, Ibaraki, Japan).

### *In vivo* antitumor study

All animal experiments were complied with the Wenzhou Medical University's Policy on the Care and Use of Laboratory Animals. Protocols for animal studies were approved by the Wenzhou Medical College Animal Policy and Welfare Committee (Approved documents: 2012/APWC/0216). Five-week-old athymic BALB/cA nu/nu female mice (18-22 g) purchased from Vital River Laboratories (Beijing, China) were used for in vivo experiments. Animals were housed at a constant room temperature with a 12 h : 12 h light/dark cycle and fed a standard rodent diet and water. SGC-7901 cells were harvested and injected subcutaneously into the right flank (5 ×10^6^ cells in 100 μL of PBS). Mice were treated by intraperitoneal (i.p.) injection of 3 mg/kg or 6 mg/kg EF24 once every other day, or by i.p. injection of 20 mg/kg 5-FU every other day, or with a combination of EF24 (3 mg/kg) and 5-FU (20 mg/kg) according to the same schedules. The tumor volumes were determined by measuring length (l) and width (w) and calculating volume (V = 0.5 × l × w^2^) at the indicated time points. At the end of treatment, the animals were sacrificed, and the tumors were removed and weighed for use in proteins activity studies.

### MDA assay

Tumors were harvested after all mice were sacrificed. The tissue samples were homogenized and sonicated in RIPA buffer on ice. Tissue lysates were then centrifuged at 12,000 g for 10 min at 4°C to collect the supernatant. The total protein content was determined by using the Bradford protein assay kit (Bio-Rad, Hercules, CA). Tumor tissue proteins were normalized according to their concentrations and subjected to MDA assay as described in the Lipid Peroxidation MDA assay kit (Beyotime Institute of Biotechnology, Nantong, China). MDA levels were detected using multimode microplate readers (SpectraMax M5, Molecular Devices, USA) at 532 nm.

### Statistical analysis

All experiments were assayed in triplicate (n = 3). Data are expressed as means ± SEM. All statistical analyses were performed using GraphPad Pro. Prism 5.0 (GraphPad, SanDiego, CA). Student's t-test and two-way ANOVA were employed to analyze the differences between sets of data. A *p* value <0.05 was considered statistically significant.
